# Food anaphylaxis to mushrooms?

**DOI:** 10.1186/2045-7022-3-S3-P151

**Published:** 2013-07-25

**Authors:** I Carrapatoso, B Bartolome, E Faria, F Ribeiro, A Segorbe Luís

**Affiliations:** 1 Imunoallergy Department, Coimbra University Hospital, Coimbra, Portugal; 2 R&D Department, Bial-Aristegui, Bilbao, Spain

## Background

We report the case of a 17- year-old male that suffered from a systemic anaphylactic episode during exercise fifty minutes after the ingestion of mushrooms cooked with wine in a restaurant. This anaphylaxis occurred at the age of 16 when the patient was playing handball indoors with a high level of humidity. The patient stopped mushrooms ingestion. There were no symptoms when the exercise occurred after the ingestion of other kinds of food, and no more anaphylactic episodes occurred. This patient suffered from persistent rhinosinusitis since the age of 10 years old.

## Methods

Skin prick tests (SPT) to commercial extracts of airborne allergens and food allergens were carried out. Prick-to-prick tests (PP) to the mushroom eaten by the patient before the reaction (*Agaricus bisporis*), total IgE and specific IgE determinations (sIgE) to airborne allergens were also performed, according to case history. The molecular mass of the IgE binding bands was calculated by means of SDS PAGE immunoblotting. In order to study the presence of cross reacting IgE in the patient serum we carried out a SDS-PAGE immunoblotting-inhibition assay using mushroom extract in solid phase and *Alternaria alternata* as inhibitor. An oral challenge test (OCT) with the restaurant mushroom dish was performed.

## Results

SPT was positive to pollen from grasses, cypress, alder and cedar as well as to *Alternaria alternata* and *Cladosporium herbarum*. PP was positive to mushroom. Total IgE level was 264 UI/ml and sIgE was positive to *Alternaria alternata* (38.8 kU/L), *Cladosporium herbarum* (4.43 kU/L), *Curvularia lunata* (3.41 kU/L), *Aspergillus fumigatus* (2.74 kU/L), and *Candida albicans* (1.71 kU/L).

Immunoblotting assays revealed two IgE binding bands of nearly 30 kDa in the mushroom extract. A total inhibition of these two IgE binding bands was detected when *Alternaria alternata* was used as inhibitor. The OCT with mushroom was negative and the patient refused an OCT with exercise after the ingestion of mushroom.

## Conclusion

Immunologic cross-reactivity between mushroom and *Alternaria alternata* was confirmed. There are a few references in the literature concerning cross-reaction between mushroom and moulds in patients with fungal allergy, particularly with rhinosinusitis. Since the OCT with mushrooms cooked in the same way as the day of the allergic reaction, was negative several factors might play a role in this reaction such as exercise performed indoors in a high level of humidity environment, with moulds.

## Disclosure of interest

I Carrapatoso: None declared, B Bartolome: Employee of 2 R&D Department, Bial-Aristegui, Bilbao, Spain, E Faria: None declared, F Ribeiro: None declared, A Segorbe Luís: None declared.

